# Erythema multiforme induced by alendronate sodium in a geriatric patient: A case report and review of the literature

**DOI:** 10.4317/jced.53653

**Published:** 2017-07-01

**Authors:** José-Alcides de Arruda, Pedro Silva, Márcio-Bruno Amaral, Fernanda Cotta, Renata Avendanho, Ricardo Mesquita

**Affiliations:** 1 Department of Oral and Maxillofacial Pathology, School of Dentistry, Universidade de Pernambuco, Camaragibe, Brazil; 2 Department of Stomatologic Sciences, School of Dentistry, Universidade Federal de Goiás, Goiânia, Brazil; 3 Department of Oral Surgery and Pathology, School of Dentistry, Universidade Federal de Minas Gerais, Belo Horizonte, Brazil

## Abstract

Erythema multiforme is an uncommon acute inflammatory disorder caused by exposure to microbes or drugs. Erythema multiforme minor typically affects only one mucosa and can be associated with symmetrical target skin lesions on the extremities. The disease usually occurs in patients in their 3rd and 4th decade of life, but can also affect children and adolescents. Alendronate sodium has been approved for the prevention and treatment of osteoporosis in postmenopausal women, but is associated with adverse events. This study reports and discusses a case of erythema multiforme minor. In addition, a literature search of articles published in PubMed-Medline was performed. The case was a 96-year-old woman who had taken alendronate. Intraoral clinical examination demonstrated hypersalivation and macrocheilia of the lower lip, associated with an ill-defined ulcer with erythematous borders measuring 20 mm in greatest diameter and covered with serofibrinous exudates. The aging of the population in developed and developing countries has increased the use of alendronate sodium to prevent osteoporosis and clinicians should be aware of possible oral adverse events associated with this drug.

** Key words:**Adverse events, erythema multiforme, therapeutics, diagnosis, alendronate.

## Introduction

Erythema multiforme (EM) is an uncommon acute inﬂammatory disorder that affects the skin, mucous membranes, or both. The disease has been classified into different variants, including the minor and major forms, which can affect the mouth alone or be associated with skin eruption in the presence or absence of oral or other mucosal lesions (1). Erythema multiforme minor typically affects only one mucosa and can be associated with symmetrical target skin lesions on the extremities. The disease usually occurs in patients in their 3rd and 4th decade of life, but can also affect children and adolescents (1,2). In contrast, EM major typically affects two or more mucous membranes and skin involvement is more variable. A severe variant of EM major is Stevens-Johnson syndrome, which is characterized by extensive skin involvement (1).

A wide range of drugs can give rise to EM and it may be difficult to clinically distinguish drug-induced EM from disease due to other causes (3,4). Previous medication use has been identiﬁed in 59% of cases of EM (5) and there has been a striking increase in the number of cases caused by drugs. More than 100 different compounds have been implicated in dermatological diseases (3).

Alendronate sodium was approved by the Food and Drug Administration for the prevention and treatment of osteoporosis in postmenopausal women and for the treatment of Paget’s disease. Alendronate belongs to a class of synthetic compounds known as bisphosphonates, which adhere strongly to hydroxyapatite crystals and inhibit bone resorption (6). The most common adverse events associated with alendronate sodium use are nausea/vomiting, nonspecific gastrointestinal events, abdominal pain, dyspepsia, diarrhea, dysphagia, and a skin rash (7).

This study reports an uncommon case of EM minor in a 96-year-old woman associated with the use of alendronate sodium. In addition, a Medline search comprising the period from 1998 to June 2016 was performed using the following keywords: erythema multiforme minor, erythema multiforme minor in oral and maxillofacial, and erythema multiforme minor by drugs.

## Case Report

**Figure 1:**
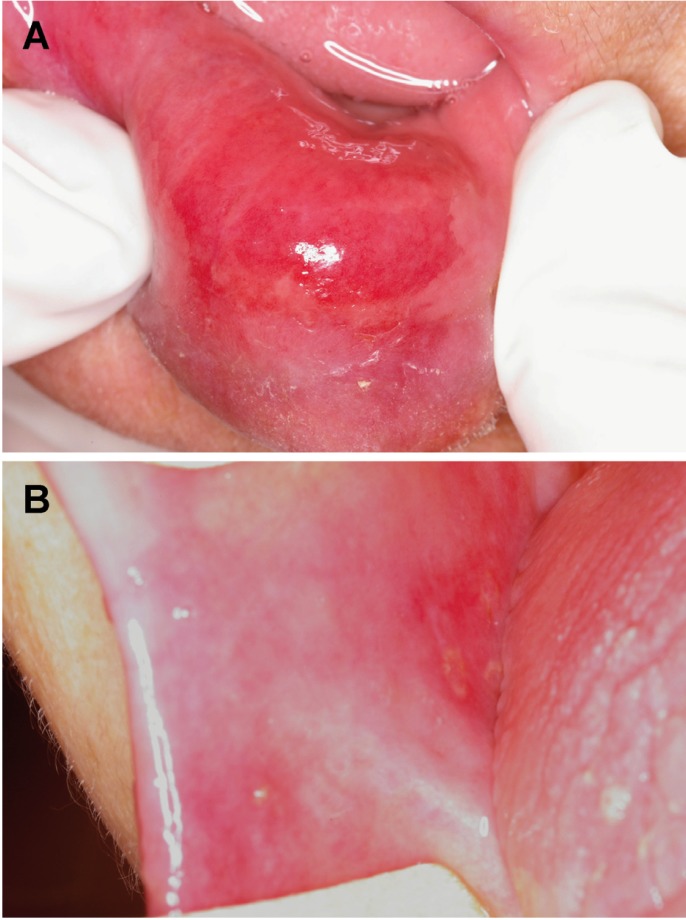
A) Well-defined ulcer located on the lower lip measuring 20 mm in greatest diameter. Note the accumulation of saliva due to sialorrhea. B) Well-defined ulcer located on the oral mucosa measuring approximately 10 mm.

A 96-year-old woman was referred by her geriatrician to the Oral Medicine Clinic, School of Dentistry, Federal University of Minas Gerais (UFMG), for evaluation of lesions on the lower lip and right cheek, which made eating difficult. Her medical history revealed osteoporotic disease, dementia syndrome, severe postural instability, congenital deafness, hypovitaminosis, peripheral venous insufficiency, and recurrent urinary tract infection. The socioeconomic history was not contributory. Intraoral clinical examination demonstrated hypersalivation and macrocheilia of the lower lip, associated with an ill-defined ulcer with erythematous borders measuring 20 mm in greatest diameter and covered with serofibrinous exudates. Other ulcers with the same features were observed in the right oral mucosa (Fig. 1 A,B). The patient was taking the following medications: B complex, calcium carbonate, anticoagulant, sulfamethoxazole/trimethoprim, and alendronate sodium. Treatment with alendronate sodium had been initiated 2 weeks before the onset of oral signs and symptoms for the prevention of osteoporotic fractures. Based on the clinical and anamnesis findings, the initial clinical diagnosis was drug-induced EM minor. Alendronate sodium, B complex and calcium carbonate were discontinued and antibiotic therapy was terminated. Analgesics, local cleaning and clinical follow-up were prescribed. The oral lesions had completely healed after 2 weeks. In agreement with the geriatrician, treatment with alendronate sodium was restarted and the same signs and symptoms appeared again 14 days later. The final clinical diagnosis was EM minor induced by alendronate sodium. The other medications were reintroduced and no signs and symptoms were observed after 4 weeks. After 24 months of follow-up, the patient shows no signs of recurrence. The patient signed the correspondent informed consent for the publication of this clinical case report.

## Discussion

Erythema multiforme is characterized by an acute onset and a wide spectrum of severity ranging from EM minor to EM major, traditionally known as Stevens-Johnson syndrome, and toxic epidermal necrolysis (Lyell’s disease) (8). The minor form of the disease manifests as ulcerations that primarily affect the oral mucosa. The oral lesions appear as erythematous plaques that undergo epithelial necrosis and progress to shallow ulcerations and erosions with irregular edges (4). In EM minor, the affected mucous membrane is limited to only one anatomical site, generally the oral mucosa (9). In the present case, the lesions occurred in a 96-year-old woman and only involved the oral mucosa. No lesions were observed at other anatomical sites and the diagnosis was the minor variant of EM.

A PubMed-Medline search was performed to identify cases of EM minor published in the English literature. The articles retrieved were analyzed regarding age, gender, cause, therapy used, outcome, and recurrence ( Table 1). As can be seen, almost every de-cade of life was affected. No gender predilection was observed and the etiology was variable. There was no established treatment protocol, but removal of the causative agent was consensus among all authors. We found no reports in the literature associating EM minor with alendronate sodium.

**Table 1:**
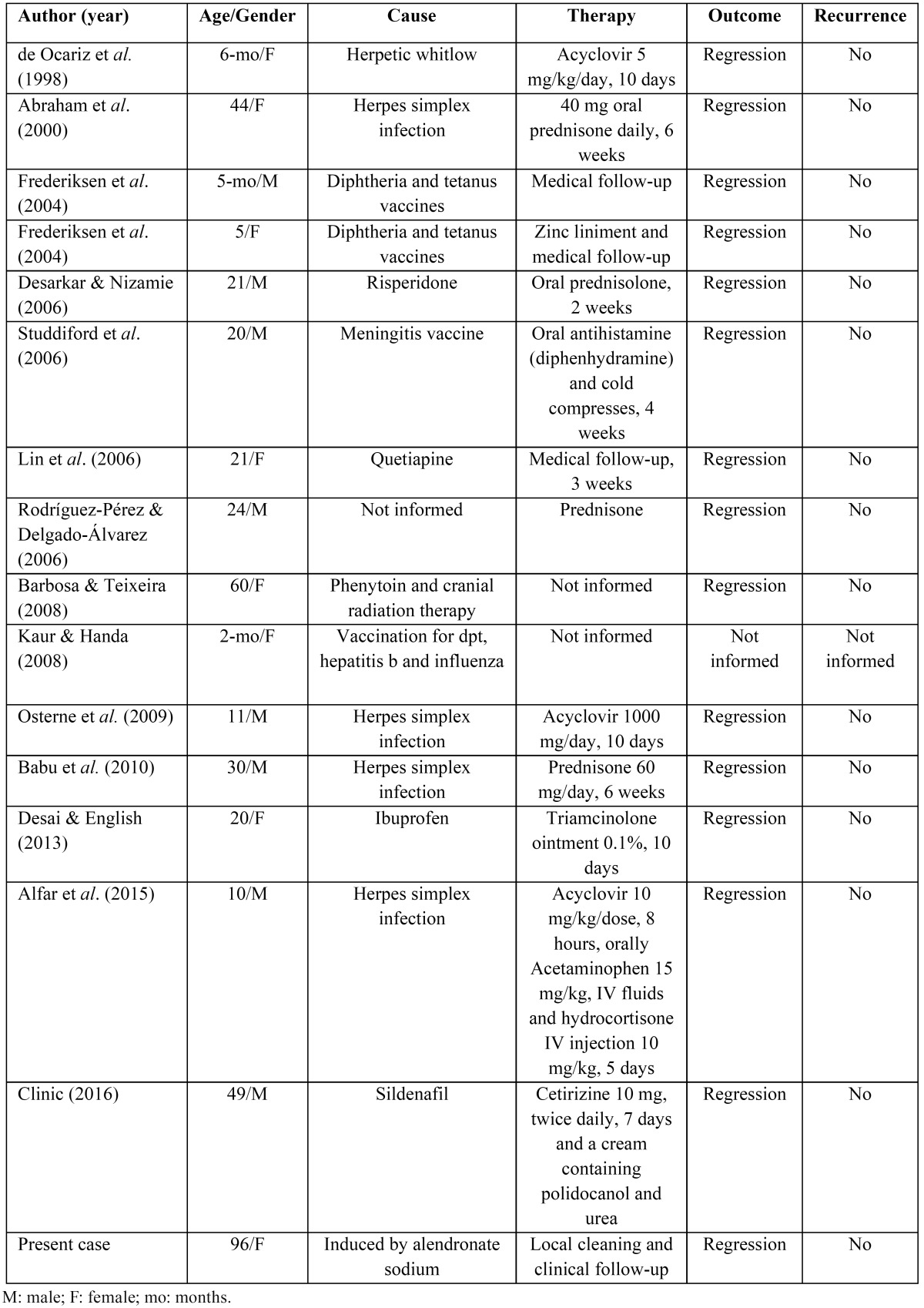
Data of erythema multiforme minor cases published in PubMed-Medline from 1998 to 2016.

The management of EM minor and major remains controversial. In the past, the use of systemic or topical corticosteroids was advocated, especially in the early stages of the disease. Although there is little good clinical evidence from controlled trials that this treatment is beneficial, it is generally used at most centers. If a causative drug is identified or suspected, treatment should be discontinued immediately. If the patient is dehydrated because of the inability to eat due to oral pain, intravenous rehydration may be necessary along with topical anesthetic agents to reduce discomfort (10-12). In the present case, regression of the lesions was observed after discontinuation of the drugs used by the patient. Although resuming treatment with alendronate sodium may have resulted in the recurrence of EM, its definitive discontinuation led to complete regression within 24 months of follow-up.

Mucosal involvement in EM minor and major varies according to severity. Patients with minimal involvement, such as painful erosions, can be treated with high-potency topical corticosteroid gel, oral antiseptic washes, and oral anesthetic solutions. Unfortunately, in some patients, extensive mucosal involvement and debilitating pain prevent sufficient oral intake. These patients require systemic glucocorticoids (e.g., 40–60 mg/d prednisone, with the dose tapered over 2-4 weeks) to reduce severity and disease duration, although there are no controlled studies to support this recommendation (11,13).

Although EM is self-limiting, lasting 2 to 6 weeks, about 20% of patients experience recurrent episodes, usually in the spring and autumn. If the disease is triggered by herpes simplex, continuous oral acyclovir or valacyclovir therapy may prevent recurrences. Persistence of EM lesions is rare. In most cases, EM is not life-threatening, except for its most severe form (10-12). Scully & Bagan (1) and Stewart *et al.* (5) recommend any precipitants of EM to be removed or treated and the causative drug to be discontinued. There is no specific treatment for EM minor. Despite controversies, corticosteroids and analgesics are the drugs most com-monly used for the management of patients with EM.

The diagnosis of EM is mainly clinical. Some lesions occurring in the skin are highly characteristic of the disease. Since there are no specific diagnostic tests for EM, the diagnosis is generally supported by a perilesional tissue biopsy and exclusion of other causes. The histopathological pattern of the perilesional mucosa in EM is characteristic but not pathognomonic. Patients typically experience prodromal symptoms such as fever, malaise, headache, cough and a sore throat that can occur one week before the onset of the disease (1). According to Ayangco & Rogers (4), the diffuse clinical appearance and oral ulcerations can be difficult to differentiate from other immunopathological disorders and the differential diagnosis must therefore include erosive lichen planus, pemphigus, pemphigoid, and linear IgA dermatosis (14). In the present case, the diagnosis was based on clinical presentation, association with drug use, and exclusion of other vesiculobullous diseases.

Although little is known about the molecular and cellular mechanisms that trigger EM, this disorder responds primarily to antigens that are induced by exposure to microbes or drugs (4). Microbial agents have been reported to trigger EM, particularly herpes simplex which is implicated in up to 70% of recurrent cases. EM is known to be related to cytotoxic T lymphocytes and specific CD8+ cells that are activated in response to antigens of drugs. Activation of the Fas membrane receptor, which is present in the cell membrane of keratinocytes, by its ligand FasL induces keratinocyte apoptosis through activation of specific enzymes called caspases (13,15).

The main drug classes associated with EM minor and major are: 1) anti-bacterial: sulfonamides (trimet-hoprim-sulfamethoxazole), aminopenicillins, cephalosporins, quinolones, and tetracyclines; 2) anticonvulsants; 3) analgesics; 4) nonsteroidal anti-inflammatory drugs, and 4) antifungals. Adverse reactions to alendronate sodium such as nausea, vomiting, abdominal pain, dyspepsia, and reflux esophagitis have been described in the literature (7,15). The clinical features of the present case support the diagnosis of EM minor caused by alendronate sodium, in agreement with Biswas *et al.* (7) who reported the case of a patient that used the same drug and developed this skin disease.

The aging of the population in developed and developing countries has increased the use of alendronate sodium to prevent osteoporosis. Skin reactions triggered by drugs can be challenging to distinguish from other vesiculobullous lesions. Knowledge of clinical practitioners about EM is important for the early diagnosis and raising awareness on the risks of indiscriminate drug use is necessary to reduce the occurrence of this disease.

## References

[1] Scully C, Bagan J (2008). Oral mucosal diseases: erythema multiforme. Br J Oral Maxillofac Surg.

[2] Katz J, Livneh A, Shemer J, Danon YL, Peretz B (1999). Herpes simplex associated erythema multiforme (HAEM): a clinical therapeutic dilemma. Pediatr Dent.

[3] Roujeau JC, Stern RS (1994). Severe adverse cutaneous reactions to drugs. N Engl J Med.

[4] Ayangco L, Rogers III RS (2003). Oral manifestations of erythema multiforme. Dermatol Clin.

[5] Stewart MG, Duncan III NO, Franklin DJ, Friedman EM, Sulek M (1994). Head and neck manifestations of erythema multiforme in children. Otolaryngol Head Neck Surg.

[6] Ragsdale AB, Barringer III TA, Anastasio GD (1998). Alendronate treatment to prevent osteoporotic fractures. Ach Fam Med.

[7] Biswas PN, Wilton LV, Shakir SA (2003). Pharmacovigilance study of alendronate in England. Osteoporos Int.

[8] Auquier-Dunant A, Mockenhaupt M, Naldi L, Correia O, Schröder W, Roujeau JC (2002). Severe cutaneous adverse reactions. Correlations between clinical patterns and causes of erythema multiforme majus, Stevens-Johnson syndrome, and toxic epidermal necrolysis: results of an international prospective study. Arch Dermatol.

[9] Al-Johani KA, Fedele S, Porter SR (2007). Erythema multiforme and related disorders. Oral Surg Oral Med Oral Pathol Oral Radiol Endod.

[10] Sanchis JM, Bagán JV, Gavaldá C, Murillo J, Diaz JM (2010). Erythema multiforme: diagnosis, clinical manifestation and treatment in a retrospective study of 22 patients. J Oral Pathol Med.

[11] Sokumbi O, Wetter DA (2012). Clinical features, diagnosis, and treatment of erythema multiforme: a review for the practicing dermatologist. Int J Dermatol.

[12] Samim F, Auluck A, Zed C, Williams PM (2013). Erythema multiforme: a review of the epidemiology, pathogenesis, clinical features, and treatment. Dent Clin N Am.

[13] French LE (2006). Toxic epidermal necrolysis and Stevens Johnson syndrome: our current understanding. Allergol Int.

[14] Pereira FA, Mudgil AV, Rosmarim DM (2007). Toxic epidermal necrolysis. J Am Acad Dermatol.

[15] Borchers AT, Lee JL, Naguwa SM, Cheema GS, Gershwin ME (2008). Stevens-Johnson syndrome and toxic epidermal necrolysis. Autoimmunity Reviews.

